# Warning signs of preschool victimization using the strengths and difficulties questionnaire: Prevalence and individual and family risk factors

**DOI:** 10.1371/journal.pone.0221580

**Published:** 2019-08-23

**Authors:** Jose-Blas Navarro, Meritxell Fernández, Núria de la Osa, Eva Penelo, Lourdes Ezpeleta

**Affiliations:** 1 Department of Psychobiology and Methodology of the Health Sciences, Universitat Autònoma de Barcelona, Bellaterra, Spain; 2 Unit of Epidemiology and Diagnosis in Developmental Psychopathology, Department of Clinical and Health Psychology, Universitat Autònoma de Barcelona, Bellaterra, Spain; Chiba Daigaku, JAPAN

## Abstract

**Introduction:**

School victimization by peers is an important social problem with serious short- and long-term consequences poorly studied at preschool ages, which can lead to school bullying without timely intervention. Longitudinal data was used to determine the prevalence of warning signs of preschool peer victimization and its individual and family risk factors.

**Methods:**

Data was obtained from 577 community preschoolers. School victimization was measured using the Strengths and Difficulties Questionnaire (SDQ) administered to parents and teachers of children at ages 4 and 5. Risk factors for the child (demographics, conduct and emotional problems, aggressiveness) and the family (maternal problems during pregnancy and early development, parenting styles, adaptive functioning and parents’ problems) were previously recorded at 3 years old.

**Results:**

Combined information from parents and teachers showed that 4.2% of preschoolers presented warning signs of victimization at ages 4 and 5. Low socioeconomic status, poor emotional control, early problems making friends and low level of parenting education in social norms at age 3 predicted later victimization at ages 4 and 5 (AUC = .78).

**Conclusion:**

Peer victimization affects a considerable percentage of preschoolers. Early detection may help to reduce the risk of escalation.

## Introduction

Peer victimization is a developmentally salient interpersonal stressor that refers to the experience of being the recipient of peer intimidation and harassment or being the target of physical, social, emotional or psychological harm from a peer [1]. As a form of aggressive behavior there is the risk of escalation and if the victimization is repeated and intentional and there exists an imbalance of power between aggressors and victim, it takes the form of bullying [2]. School victimization is an important social problem with serious short-term consequences for victims’ physical and psychological health and negative long-term effects on their future psychosocial adjustment as adults.

The cumulative consequences of early victimization may emerge at later points in children’s school careers and have been well documented [3,4]. In cases where the victimization becomes chronic, the consequences may have an even greater effect on children’s achievements and learning [5]. Peer victimization has been associated with emotional and behavioral problems over time that are beyond the child’s pre-existing difficulties [6], with children who are victimized at risk of a wide range of adjustment difficulties that include: self-harm [7], loneliness, social avoidance, self-blame [8], suicide ideation [9], anxiety and depression [10], social maladjustment, loneliness [11], lack of close peer relationships [12], low self-esteem [13], poor physical health, psychosomatic complaints, behavioral dificulties and deficient emotional adjustment [13,14]. Other important findings show there is also a potential significative impact on adult adjustment outcomes [15] such as problems with doing housework and managing money [16], trouble forming new social relationships, less social support, poorer family functioning and lower levels of education [17].

Early diagnosis and timely treatment of these problems at a young age will not only improve children’s adaptation at school and their emotional and social development [18], but will also help to prevent their impact in later years.

### Preschool and peer victimization

Most of the research about peer victimization has focused on middle childhood or adolescence. Beyond the home, school is usually the main environment where children’s difficulties with social interactions with their peers can be primarily detected and subsequently assessed by adults and professionals [18]. During preschool and kindergarten, children learn how to build and maintain friendships, and they form opinions about who they like or dislike and they establish groups of consistent play partners [19]. Experts on child development strongly agree on the importance of acquiring cognitive and social-emotional competencies at the pre-school stage [20].

The manifestation of peer victimization in the preschool years is similar to that of school-age children in many ways but differs in others depending on the child’s development of social cognition [21]. Younger students usually tend to resort to physical aggression, possibly because they have not yet developed the required sophistication of verbal or social skills to obtain what they want. With maturation, children start utilizing verbal skills to engage in more subtle forms of verbal aggression [22,23] and/or develop more complex social skills such as assessing and manipulating social situations, and they can also engage in indirect forms of aggression [22]. For these reasons it is important to study peer victimization from preschool ages.

### Prevalence and measure

Although the most severe forms of peer victimization seem to have reduced in many countries over the last few years [24], efforts to detect and prevent this type of aggressive behavior in schools are still essential. There is an abundance of large-scale studies on elementary and high school children [12,25], but less studies have been carried out on preschoolers. The literature shows that even at these early ages peer victimization occurs frequently and is associated with poor psychosocial well-being [26]. Ilola et al. [27] found a 4% six-month prevalence of frequently bullied children as reported by the parents of a sample of 931 4-year-olds who attended pediatric checkups. In another large population-based study of 5- to 6-year-olds that used reports from parents and teachers, a similar 4% of the children were victims [10].

A problem like peer victimization, which remains hidden in most cases, requires information from as many sources as possible. The Strengths and Difficulties Questionnaire (SDQ) [28] is a tool with good psychometric properties that is widely used to screen for psychiatric disorders and its version for children aged 3 to 4 years old has been utilized at preschool age [29,30]. The SDQ has versions for parents and teachers to account for discrepancies between informants, since different informants observe children’s behavior in different settings [31]. Parents and teachers are the ones who spend most time with pre-school aged children. Given the preventive aim of early detection, both reports are indispensable under the Attribution Bias Context Model [32], which posits that informant discrepancies are indicative of cross-contextual variability in children’s behavior and informants’ perspectives on this behavior. The SDQ includes a peer problems scale, which contains a specific item for peer victimization. If this questionnaire is widely used and registers the presence of peer victimization, it could be used for identifying early warning signs of problematic behaviors indicating that the child is a target of aggressive victimization behaviors, and for studying their risk factors. A warning sign is an observable manifestation that hints of the existence of something that may impact negatively on the person, in this case peer victimization.

### Risk factors

To help designing preventive and intervention strategies against peer victimization, it is crucial to determine the risk factors that enable the prediction of the onset of victimization to facilitate an early identification of infants at risk of becoming victims [33]. Several longitudinal studies have identified factors that relate children’s personal characteristics and their home environment with an increased risk of being victimized [34].

The social-ecological model posits that peer victimization involvement is determined by the multiple systems in which youth are embedded [35]. First introduced in the late 1970s [36], this ecological model was initially applied to child abuse [37] and subsequently to youth violence [38]. The model explores the relationship between individual and contextual factors and considers violence as the product of multiple levels of influence on behavior [39]. Understanding how these factors are related to peer victimization is one of the essential steps in the public health approach to preventing this violence [40].

The first level of the ecological model focuses on the intrinsic characteristics of the individual that might increase the likelihood of their either becoming a victim or a perpetrator of violence, and the guiding assumption for these factors is that victimized children behave in ways that invite or reinforce attacks against them [41]. It has been suggested that aggressors “often have a positive outlook on the use of violence to solve problematic situations or get what they want” [42]. This follows the theoretical assumptions underlying proactive aggression. Bullies can be quite adept at identifying victims who will either not retaliate or will be ineffective in their efforts to retaliate, making them more desirable targets [43]. Some children may have temperamental, behavioral and cognitive characteristics that evoke aggressive behavior from others and make them more prone to victimization.

Among children and adolescents, longer exposure to peer victimization has been associated with higher emotional sensitivity, anger, fear and internalizing and unstable affective temperaments [44]. Similarly, psychopathology may contribute to victimization. Over time internalizing (withdrawal, anxiety/depression and hovering peer entry style) and externalizing (aggression, argumentativeness, dishonesty, pushy peer entry style and disruptiveness) behavior problems are associated with victimization [45]. However, prospective studies of young children that examine such antecedent effects are largely lacking [46]. Few studies have tackled the cognitive characteristics of the victimized children. In a Brazilian sample was found that 10- to 11-year-old child peer victims presented difficulties in executive functioning and showed lower cognitive flexibility [47]. Previous studies regarding victimization and ethnic and/or racial differences have produced mixed or inconclusive results [48]. Some studies report that minority youth are more likely to be victimized [49], whereas others have found that the percentage of immigrants and the racial variability of school children do not seem to be risk factors for being victimized [50].

The second level of the ecological model is related to the family environment and explores how proximal social relationships, for example relations with family members, increase the risk of victimization [40]. The impact and relation of family characteristics as risk factors of victimization has gained increased attention in recent years. It has been shown that parental characteristics and adverse lived experiences may have an impact on children, facilitating peer victimization [51]. Several studies have shown that peer victimization is associated with inconsistent, punitive, hostile and/or abusive parenting, high negative expressiveness and high levels of family conflict or violence [52,53]. Also, children growing up in low or mean socioeconomic status (SES) families are at increased risk of victimization [54]. Furthermore, victims from schools in low-SES communities or with a large proportion of students from low-SES families suffer worse long-term mental illness as consequence of the victimization compared to victims from richer families [54]. Additionally, severe prenatal family adversity significantly increased the risk of peer victimization at school [55]. Also, children whose relatives were abused during childhood have an increased risk of being bullied, which may indicate an intergenerational transmission of maltreatment [56]. Last, parental psychopathology also affects the child’s risk of being victimized through complex pathways, such as genetic, neurobiological (e.g., dysregulated stress systems) and social (e.g., modelling) ones [57].

### Purpose of the study

The present investigation is based on the premise that children’s behavioral attributes and family factors contribute to their victimization, and has the aim of identifying the children with an increased risk of becoming a victim at the youngest possible age to facilitate timely prevention of the problem. Awareness should be raised about how the instruments at use allow us to identify the problem and the characteristics of the identified children to determine their usefulness in screening for peer victimization [58].

Using a prospective longitudinal design, the present study seeks to contribute new knowledge about the early individual and family risk factors of being peer victimized during the preschool period and seeks to obtain an estimate of the prevalence of these warning signs of peer victimization at 4 to 5 years old, using a widely used screening instrument (SDQ). In alignment with Bronfenbrenner’s ecological model [36], 35 individual and family factors were initially selected to identify which were the most predictive of peer victimization. Selection of the final set of risk factors was made through a predictive modelling approach.

## Materials and methods

### Study design and procedure

The data was part of a large-scale longitudinal study of behaviour problems in preschool children from age 3, who were screened for behaviour problems and followed up annually until age 9 (the design procedure is detailed in [59]). The two-phase design used involved selecting first a random sample of 2,283 children from the census of in-school 3-year-old preschoolers in Barcelona (Spain) during the 2009–10 academic year (N = 13,578 children). The sample size was determined so that the study was able to detect associations between exposure to different risk factors and the presence of psychopathology equal to or greater than OR = 2.0, accepting an alpha risk of 5% and a statistical power of 80%. Epidemiological studies indicate that the prevalence of psychopathology in children and adolescents (range of ages from 6 to 16 years) of the general population is approximately 15% (p = 0.15) [60]. Likewise, a multiple correlation between covariates of 0.4 (R^2^ = 0.16) is assumed. Some corrections were applied to the obtained sample: a) correction for homogeneity of children within the same classroom (random clustered sampling) increasing the sample by 20%; b) Correction because double stage design. It was established that screening would classify children into two equally sized groups (50%). Because only 30% of the group of children with negative screening will be followed, it is necessary to proportionally increase the initial number of children to be recruited; and c) Correction for attrition due to refusal of the school to continue participating (assuming 20% of centers will abandon) and due to refusal of subjects, assuming 50% of families will leave the study (as indicated by similar follow-up studies carried out in advanced countries). Exclusion criteria included the child having a generalized developmental disorder, the parents having reading problems or not understanding neither Spanish nor Catalan languages and having planned a move outside Barcelona. All the parents of the initially selected pupils were invited to participate in the study, from which 1,341 families (58.7%) agreed to participate. There were no sex or socioeconomic differences (p = .95 and p = .182) between those who agreed to participate and those who declined, although more participants than refusals attended private schools (p < .001).

**Fig 1:**
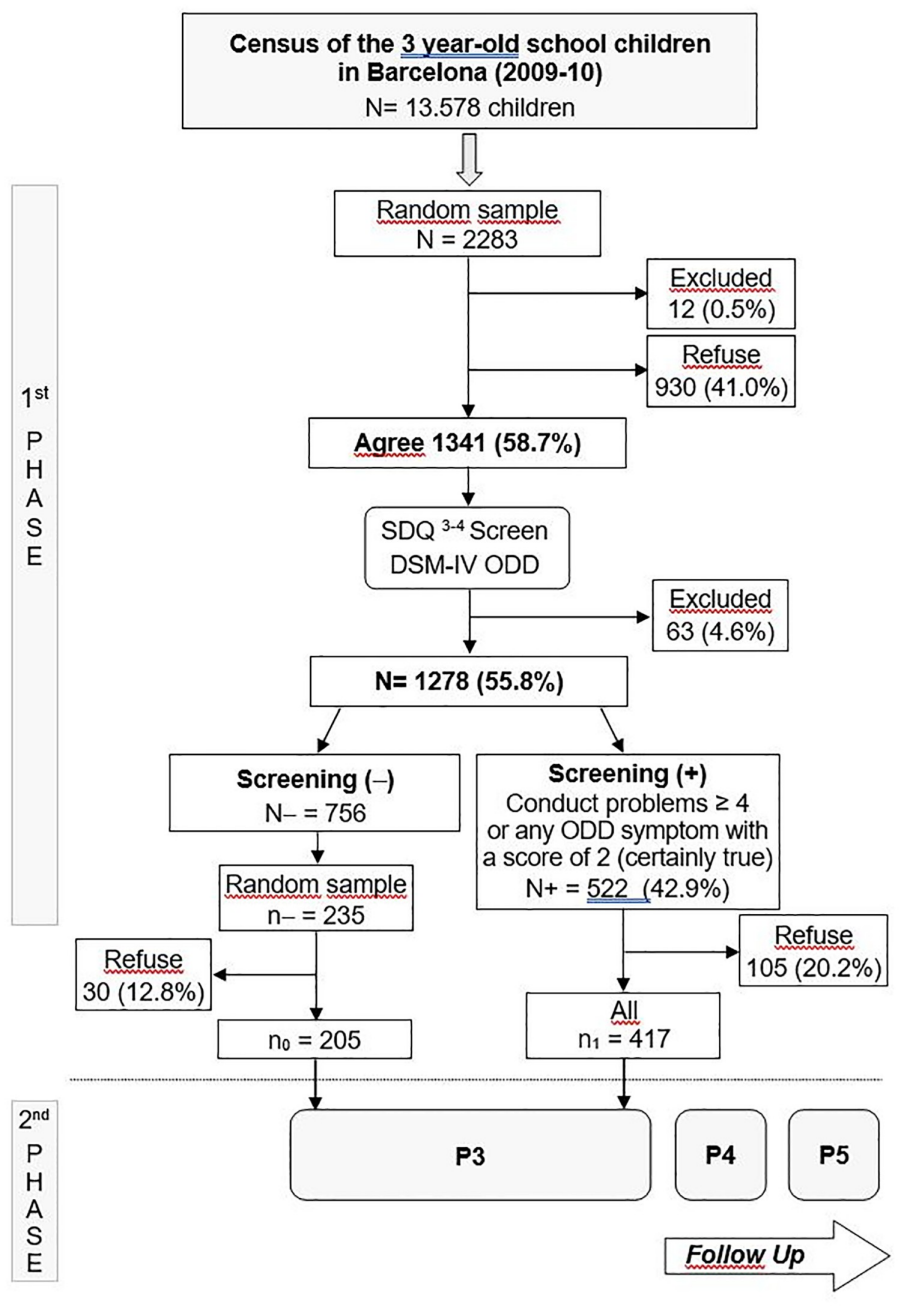
Sampling procedure of the study.

The second phase of the sampling consisted in a screening of behavioral problems carried out using the SDQ conduct problems scale [28] (see below) plus four symptoms of DSM-IV oppositional defiant disorder (ODD) (the principal disorder of interest in the original study) not present in the questionnaire (deliberately annoys people, blames others, touchy, angry and resentful). To ensure the presence of children with behavior problems in this second phase, all of those who screened positive for behavioral problems and a random sample of around 30% of children who screened negative (the number of children needed in the negative screening score was calculated to guarantee statistical power) were invited to continue. The final second phase sample included 622 families (10.6% of those invited declined to participate). No differences were found on comparing participants and refusals either by sex (p = .820) or school type (p = .850). The families were interviewed at the school. Interviewers were previously trained and were blind to the children’s screening group. The teachers were asked to complete the questionnaires before the end of the academic year. The Fig 1 shows the sampling procedure.

The research project was approved by the Ethics Commission on Animal and Human Experimentation from the Autonomous University of Barcelona. The parents of the participants children were informed and gave written consent to participate in the study.

### Instrumentation

#### Outcome measures at ages 4 and 5

Strengths and Difficulties Questionnaire for ages 3–4 (SDQ^3-4^) [28] for parents and teachers. It is a screening questionnaire for behavioural and emotional problems applied for the purposes of this study when the children were 3 (parents version for control variables), 4 and 5 years old (parents and teachers for the outcome variable). Item 19 - ‘Picked on or bullied by other children’, coded as 0: not true; 1: somewhat true; 2: certainly true—answered by parents and teachers was selected as outcome. We conceptualized that if parents or teachers scored 1 or 2 on this item at both follow-ups at ages 4 and 5 this would be a warning sign for investigating the child’s possible victimization; that is, if the problem as reported by any of the informants was sustained over time, the case would be considered as victimized. Good psychometric properties in the sample have been reported [61], with Cronbach’s alpha = .73 for parents and .82 for teachers at age 3, and slightly better values for ages 4 and 5.

#### Predictive measures at age 3

Child Behavior Checklist (CBCL/1½-5) [62]. It is a questionnaire of behavioral and emotional problems reported by parents through 100 items with 3 response options (0: not true, 1: somewhat/sometimes true, 2: very true/often true). Internalizing and externalizing empirical scales, plus the DSM-oriented scales depressive, anxiety, developmental, attention deficit/hyperactivity and oppositional defiant problems, were used for the analyses. Higher scores indicate higher symptomatology.

Behavior Rating Inventory of Executive Function—Preschool Version (BRIEF-P) [63]. It is a standardized questionnaire that assesses behaviors reflecting executive functions in daily life in preschool children. The instrument consists of 63 items on a 3-point scale (1: Never, 2: Sometimes, and 3: Often). Two dimensions which may be associated with social problems, Inhibit (I) and Emotional Control (EM), were used and higher scores indicated greater difficulties. Teachers answered the questionnaire when the children were aged 3. Good psychometric properties have been reported [64].

Children’s Aggression Scale (CAS) [65]. It is a questionnaire that assesses aggressive behavior with 22 items on a 5-point Likert-type scale (0: never to 4: many days). The total score of the teachers’ responses was used as a global index of aggressive behavior. Higher scores indicate greater aggressive behavior.

Children’s Behaviour Questionnaire for ages 3–7 years (CBQ3-7) [66]. It is a questionnaire that measures reactive and self-regulative temperament, with 94 items answered by parents on a 7-point Likert-type scale, ranging from 1 (extremely untrue) to 7 (extremely true). The 3 broad dimensions of temperament: negative affectivity, effortful control and surgency were considered. Higher scores indicate more marked traits. The reported psychometric properties are good [67].

Schedule for Risk Factors (SRF) [68]. It is a computerized structured interview conceived as a compendium of the potential areas of risk of psychopathology that should be evaluated in children. Demographic information including SES [69], pregnancy events, early development and history of abuse suffered by family members were used for this analysis. These risks factors were registered as present/absent as reported by parents. The reported psychometric properties are good [70].

Alabama Parenting Questionnaire for Preschool (APQ-Pr) [71]. It was used to assess parenting practices. It contains 24-item (1: Never to 5: Always) which measures three dimensions of parenting: positive discipline techniques, inconsistent parenting and punitive parenting. Two additional scales extracted from the Evaluation des Pratiques Educatives Parentales–Preschool and Primary School Form (EPEP–PPSF) [72], norms (6 items, labelled as Rules in the EPEP-PPSF) and autonomy (3 items), were added to the instrument with the same response format. In the original EPEP-PPSF, items assessing rules and autonomy emerged as two separated factors and jointly with items assessing positive parenting emerged as a second-order factor of Support; the authors also found low but significant positive correlations between these three measures and desirable traits in the child’s personality. In our sample, an exploratory factor analysis for categorical items conducted with MPlus8.1 showed that the 9-item and 2-factor model with Geomin rotation (χ^2^ [17] = 108.0, comparative fit index [CFI] = .990, root mean square error of approximation [RMSEA] = .091) for Norms and Autonomy items fitted better than a 9-item and 1-factor model (χ^2^ [9] = 721.7, CFI = .925, RMSEA = .212); scree test based on eigen values also supported the retention of two factors. All items had salient loading in the expected factor (λ ≥ .65; *p* ≤ .001). Moreover, in our sample a confirmatory factor analysis conducted with MPlus8.1 for the whole set of items, that is considering a 33-item and 5-factor model, showed acceptable fit (χ^2^ [485] = 1112.8, CFI = .945, RMSEA = .048), all factor loadings being at least moderate and statistically significant (λ ≥ .34; *p* ≤ .001); as expected, the higher values for factor correlations were found between norms, autonomy, and positive discipline techniques (*r* between .35 and .57). For all five measures used in the present study, higher scores indicate higher parental practices in the direction of the label of the scale.

Adult Self-Report (ASR) [73]. It is a questionnaire of dimensional psychopathology for adults aged 18–59. It contains 126 items (0: not true, 1: somewhat or sometimes true, and 2: very true or often true) and was answered by the mothers. DSM-oriented scales were analyzed. Higher scores indicate higher symptomatology.

Second column of Tables 1 and 2 show the Cronbach’s alpha values of the measures in the sample.

**Table 1:** Descriptive statistics of individual predictors at age 3 and results of logistic regression models predicting warning signs of peer victimization at ages 4–5.

Individual predictors	Cronbach’s α	Warning signs		Logistic regression	
		No	Yes		
		*n* (%) or Mean (*SD*)		*OR*	95% CI *OR*
Demographics (*n* = 577)					
Sex (male)		277 (50.1%)	12 (50.0%)	1.11	0.42; 2.93
Ethnicity (non-Caucasian)		58 (10.5%)	1 (4.2%)	0.18	0.02; 1.58
SES (low or medium-low)		110 (19.9%)	12 (50.0%)	5.15*	**1.91; 13.94**
CBCL 1½-5 (*n* = 573)					
Internalizing	.83	7.2 (5.8)	10.5 (7.6)	**1.08**	**1.01; 1.17**
Externalizing	.85	10.5 (6.1)	12.3 (7.4)	0.99	0.92; 1.07
Depressive problems	.51	1.9 (2.0)	2.5 (1.9)	1.01	0.80; 1.27
Anxiety problems	.65	3.0 (2.6)	4.3 (3.1)	1.05	0.82; 1.33
Developmental problems	.65	2.7 (2.5)	4.5 (2.8)	**1.20**	**1.01; 1.42**
ADH problems	.74	4.1 (2.6)	4.7 (2.8)	1.05	0.83; 1.33
Oppositional defiant problems	.74	3.2 (2.2)	3.4 (2.5)	0.90	0.66; 1.24
BRIEF-P (*n* = 575)					
Inhibit	.93	23.1 (7.0)	24.6 (7.8)	0.99	0.93; 1.05
Emotional control	.88	12.1 (3.5)	14.0 (4.4)	1.14*	**1.03; 1.26**
CAS (*n* = 563)					
Total score	.82	265.2 (20.5)	271.8 (23.7)	1.01	0.99; 1.03
CBQ (*n* = 574)					
Surgency	.74	4.3 (0.8)	4.2 (0.7)	0.93	0.54; 1.60
Effortful control	.79	5.2 (0.6)	5.5 (0.7)	2.01	0.87; 4.67
Negative affectivity	.71	3.8 (0.7)	3.9 (0.7)	1.40	0.77; 2.56

Note: In brackets, for categorical predictors: risk category; in bold, results for predictors with *p* < .10

* *p*-value remained below .10 after False Discovery Rate

ADHD: attention-deficit/hyperactivity disorder; SDQ: Strengths and Difficulties Questionnaire; CBCL: Child Behavior Checklist; BRIEF-P: Behavior Rating Inventory of Executive Functioning-Preschool version; CAS: Children’s Aggression Scale; CBQ: Children’s Behavior Questionnaire.

**Table 2:** Descriptive statistics of family and early predictors at age 3 and results of logistic regression models predicting warning signs of peer victimization at ages 4–5.

Family and early predictors	Cronbach’s α	Warning signs		Logistic regression	
		No	Yes		
		N (%) or Mean (*SD*)		*OR*	95% CI *OR*
Risk Interview (*n* = 567)					
Pregnancy					
Maternal emotional problems (*yes*)		105 (19.3%)	10 (41.7%)	**2.31**	**0.86; 6.21**
Living with conflict (*yes*)		50 (9.2%)	3 (12.5%)	0.74	0.14; 3.76
Economic problems (*yes*)		16 (2.9%)	5 (20.8%)	**5.11**	**1.33; 19.68**
Early Development					
Postpartum depression (*yes*)		113 (20.8%)	9 (37.5%)	2.26	0.82; 6.17
Clumsy child (*yes*)		64 (11.6%)	6 (25.0%)	**2.45**	**0.85; 7.07**
Difficulty making friends (*yes*)		47 (8.5%)	5 (20.8%)	4.95*	**1.47; 16.63**
History of abuse					
Relative abused in childhood (*yes*)		54 (9.8%)	6 (25.0%)	6.87*	**1.69; 27.89**
Biological mother abused (*yes*)		36 (6.5%)	3 (12.5%)	0.24	0.04; 1.32
APQ-Pr (*n* = 561)					
Positive parenting	.75	40.8 (4.2)	41.2 (3.5)	1.09	0.98; 1.22
Inconsistent parenting	.62	6.9 (3.1)	6.8 (3.2)	0.94	0.80; 1.11
Punitive parenting	.42	3.7 (1.9)	3.9 (2.3)	1.12	0.90; 1.40
Norms	.88	22.4 (2.5)	20.1 (4.4)	0.74*	**0.66; 0.83**
Autonomy	.81	9.5 (2.1)	9.4 (2.0)	1.11	0.88; 1.39
ASR-DSM Mother (*n* = 517)					
Depressive	.77	3.2 (3.2)	5.3 (4.2)	1.05	0.88; 1.24
Anxiety	.64	5.5 (2.4)	6.8 (2.6)	1.08	0.87; 1.35
Somatic	.69	1.1 (1.7)	2.9 (3.9)	**1.28**	**1.02; 1.62**
Avoidant personality	.66	2.2 (1.9)	3.4 (2.6)	1.18	0.91; 1.52
ADHD	.75	3.9 (3.2)	4.8 (3.9)	0.86	0.67; 1.09
Antisocial personality	.55	2.2 (2.1)	3.1 (2.5)	1.09	0.89; 1.33

Note: In brackets, for categorical predictors: risk category; in bold, results for predictors with *p* < .10

* *p*-value remained below .10 after False Discovery Rate.

APQ-Pr: Alabama Parenting Questionnaire for Preschool; ASR-DSM: Adult Self-Report DSM scales; ADHD: attention-deficit/hyperactivity disorder

### Statistical analysis

The software used for statistical analysis was Stata 15 for Windows. To reflect population prevalence (as final participant selection was conditioned by the presence/absence of behavior problems), the analyses were weighted by the inverse probability of being selected in the second phase of the sampling design.

A two-stage modelling procedure [74] was used to determine the best predictive model of 4- to 5-year-old warning signs of peer victimization from the 35 predictors pre-selected at age 3 according to the literature. First, several independent binary logistic regression models were estimated, grouping the predictors by content (demographics, individual and family risk factors). Because of the large number of predictors included in this first stage, the false discovery rate (FDR) [75] was applied to control the Type I error and to know which predictors remained significant after correction. Second, predictors with an FDR-corrected significance p-value below .10 were selected and a backward stepwise binary logistic regression model was conducted. Predictors from this model with p-values below .05 were used to calculate the area under the ROC curve (AUC), which indicated the proportion of correct predictions of warning signs of victimization achieved with the selected predictors. AUC values above .75 are usually considered as indicating a good prediction capability. This final model included as covariates parents’ and teachers’ answers to item 19 of the SDQ (“Picked on or bullied by other children”) at age 3 to adjust the odds-ratios of victimization at baseline. The predictive capability of the model was calculated through Pseudo-R^2^ [76] and translated in terms of ordinary R^2^ using the same author’s approach.

Since no cross-validation weighted commands have been developed for Stata, the final predictive model was validated using a repeated random sub-sampling cross-validation procedure [77], which produces a summary measure of loss of prediction.

The Hosmer-Lemeshow goodness-of-fit test was verified for each logistic model and the absence of outliers and influential observations was also confirmed [78].

## Results

A total of 602 children were available at age 4 (96.8% from the 622 children starting the study at age 3) and 577 (92.8%) remained at age 5. Participants and drop-outs at age 5 were statistically equal in sex (p = .297), but participants had a higher socioeconomic status (SES) than drop-outs (p = .002). Parents’ information was obtained from mothers (67.6%), fathers (7.6%) or both (24.8%), with a mean age of 37.6 years. Only 5 out of 577 teachers were male. Table 3 shows the demographic characteristics of participants for the total sample and separately by absence/presence of victimization warning signs.

**Table 3:** Demographics descriptive for total sample at age 3 and grouped by warning signs of peer victimization at ages 4–5.

	Total sample	Warning signs = No	Warning signs = Yes	χ^2^ or Fisher exact’s test
	*N* = 577	*N* = 553	*N* = 24	
	*N* (%)	*N* (%)	*N* (%)	*p*
Sex				.993
Girls	288 (49.9%)	276 (49.9%)	12 (50.0%)	
Boys	289 (50.1%)	277 (50.1%)	12 (50.0%)	
Ethnic group				.652
Caucasian	518 (89.8%)	495 (89.5%)	23 (95.8%)	
Hispanic-American	33 (5.7%)	33 (6.0%)	0 (0%)	
Other	26 (4.5%)	25 (4.5%)	1 (4.2%)	
Socioeconomic status				.013
High	192 (33.3%)	187 (33.8%)	5 (20.8%)	
Medium-High	177 (30.7%)	171 (30.9%)	6 (25.0%)	
Medium	86 (14.9%)	85 (15.4%)	1 (4.2%)	
Medium-Low	93 (16.1%)	84 (15.2%)	9 (37.5%)	
Low	29 (5.0%)	26 (4.7%)	3 (12.5%)	

### Prevalence of warning signs of peer victimization

According to the answers given to SDQ-item 19 (“Picked on or bullied by other children”) by the 577 participants remaining in the study at age 5, and applying our definition of warning signs of peer victimization (if answers were 1 or 2), the parents reported warning signs at one follow-up (4 or 5 years old) for 15.9% of preschoolers and at both follow-ups for 3.0% of preschoolers. Teachers reported comparable results, with 16.4% of children presenting warning signs at only one age and 1.4% at 4 and 5 years old. To minimize lack of sensitivity, a child was considered to display warning signs of peer victimization if parents or teachers reported the item positively at both ages 4 and 5. On applying this definition, 24 children (4.16%, 95% CI [2.68, 6.13]) were classified as displaying warning signs of peer victimization. Chi-square tests revealed that there were no differences by sex or ethnicity in the prevalence of warning signs of peer victimization, but the risk increased as SES decreased (Table 3).

### Initial selection of risk factors of warning signs of peer victimization at 4 to 5 years old

Table 1 presents descriptive statistics for the individual characteristic predictors (demographics, dimensional psychopathology, executive functioning, aggressive behavior and temperament) for children with and without warning signs of peer victimization separately; the last three columns show the results of the first stage of the modelling procedure, consisting of the estimation of several independent logistic regression models to predict warning signs of peer victimization at 4 to 5 years old (one model for each set of predictors: demographics, dimensional psychopathology, etc.). Four individual attributes showed significant odds-ratios: low or medium-low SES, CBCL-internalizing, CBCL-developmental problems and BRIEF-P emotional control difficulties. After applying the FDR correction to control the increment of the type I error due to the high number of statistical tests performed, two predictors remained significant at .10 value: SES and BRIEF-P emotional control.

Similarly, Table 2 shows descriptive statistics for the family and early predictors (pregnancy, early development, history of abuse, parenting practices and maternal psychopathology). The last three columns show the odds ratios obtained from the first phase of the modelling strategy, estimating independent logistic regression models to predict warning signs of victimization at 4 to 5 years old from each set of predictors (pregnancy, early development, etc.). Five significant odds ratios were found: economic problems during pregnancy, child’s early difficulties in making friends, a history of childhood abuse among relatives, lower norms and higher ASR-somatic problems as reported by the mother. Statistical significance remained below .10 after FDR correction for three predictors: difficulties in making friends, childhood abuse among relatives and lower norms.

### Final predictive model with risk factors of warning signs of peer victimization at ages 4 to 5

The five predictors with FDR-corrected p-values under .10 (two individual and three family predictors) were selected as the maximum logistic regression model in the second stage of the modelling procedure. To select the significant predictors from these five a backward stepwise regression was applied, which retained four significant predictors (Table 4). This final model was adjusted by the baseline presence/absence of item 19 reported by both parents and teachers. The resulting odds ratios show that the risk of presence of warning signs of peer victimization at 4 to 5 years old increased for children with a low or medium-low SES (p = .001), higher scores in BRIEF-P emotional control (p = .004), difficulties in making friends (p = .015) and parenting practices characterized by lower scores in APQ norms (p < .001).

**Table 4:** Final logistic regression model with selected predictors at age 3 of warning signs of peer victimization at ages 4–5.

Final model–*Pseudo R*^*2*^ = .22 (*n* = 565)	Logistic regression*	
	*OR*	CI 95% *OR*
Socioeconomic status (*low or medium-low*)	**5.39**	1.98; 14.66
BRIEF-P emotional control	**1.15**	1.04; 1.26
Difficulty in making friends (*yes*)	**4.63**	1.35; 15.83
APQ-Pr-Norms	**0.80**	0.71; 0.91

Note:

*Adjusted by parents and teachers’ answers to SDQ item 19 (victimization at 3 years-old);

in bold, results for predictors with *p* < .05

SDQ: Strengths and Difficulties Questionnaire; BRIEF-P: Behavior Rating Inventory of Executive Functioning-Preschool version; APQ-Pr: Alabama Parenting Questionnaire for Preschool

**Fig 2:**
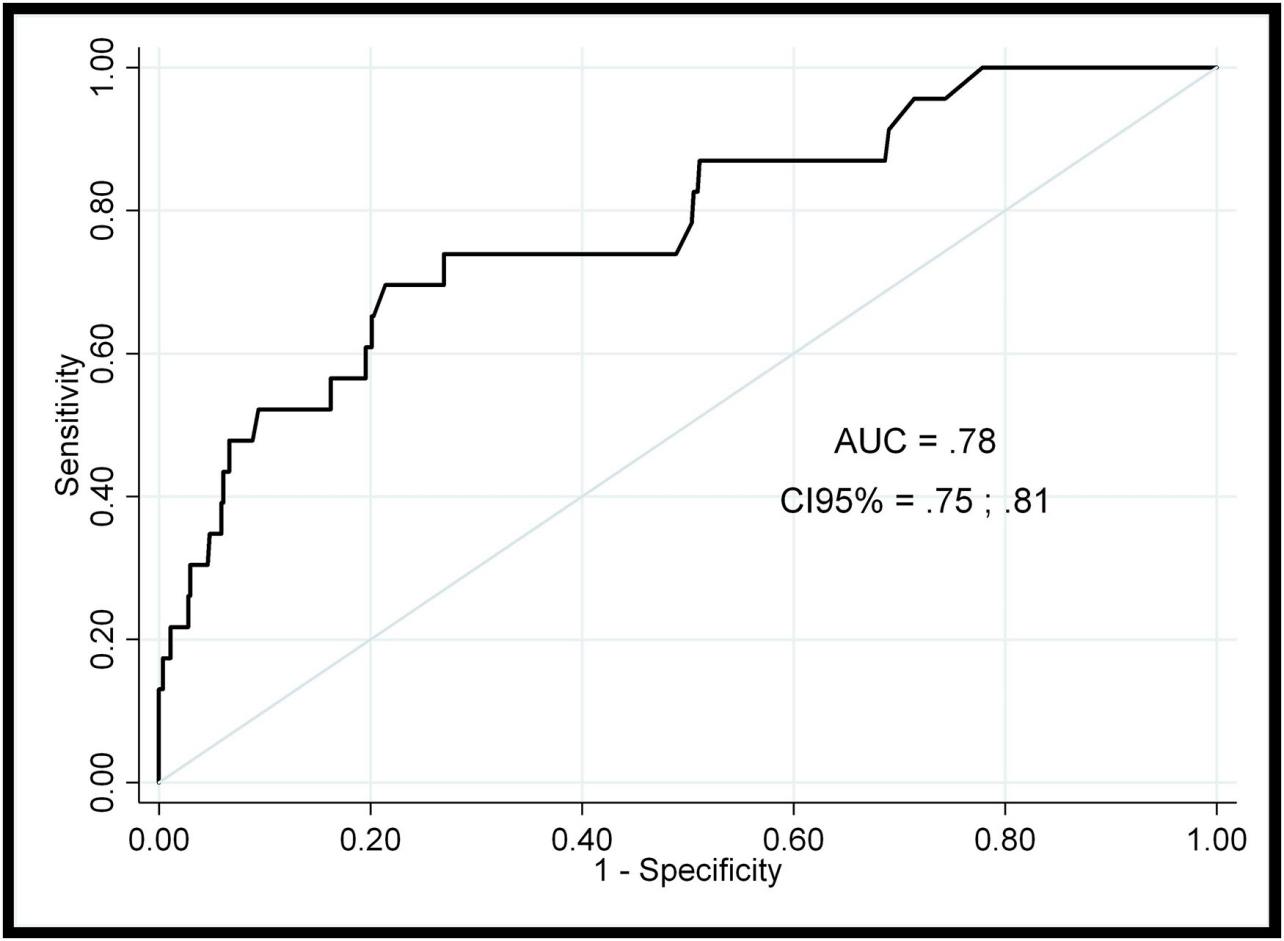
ROC curve and AUC for the predictive final model of warning signs of peer victimization at 4–5 years-old.

This final multiple model, which included four terms plus the two adjusting terms, obtained a Pseudo-R^2^ = .22, which according to [76] is approximately equivalent to an R^2^ = .50 of a linear regression. Fig 2 shows the corresponding ROC curve, which reflects the accuracy of these four variables to predict the presence of warning signs of victimization. The AUC = .78 (95% CI [.75, .81]) is statistically better than a classification made by chance (p < .001) and could be considered as almost excellent [74]. Generalizability of the predictive model could be considered acceptable as loss of prediction obtained through repeated random sub-sampling cross-validation was only .044 in terms of AUC.

## Discussion

The purpose of this study was to report on the prevalence and risk factors of warning signs of preschool victimization as assessed by the SDQ, a widely used screening questionnaire reported by parents and teachers. According to the endorsement of item 19 (“Picked on or bullied by other children”), 4.2% of 4- to 5-year-old in-school preschoolers suffered persistent victimization. Although few studies have reported the prevalence of warning signs of victimization in preschoolers, our estimation of prevalence (4.2%) is in line with that reported by previous studies [10,27,79] for children of similar ages in other cultures using one item of a questionnaire for assessing the rates of bullying. Our assessment of victimization was also done with one item of a widely used instrument, the SDQ.

Given that aggressive behavior tolerance at any age should be near 0, our results indicating a prevalence of warning signs of victimization in preschoolers equal to 4.2% showed that from preschool age there was a considerable number of children persistently exposed to being picked on or bullied by other children. The adverse consequences of preschool victimization are manifested in children and adolescents in a deteriorated mental health (depression, anxiety, suicidal ideation and suicide attempts) and in the increase of psychosocial problems [80].

A variety of individual and family factors at age 3 increased the risk of parents and teachers reporting persistent victimization: pertaining to a low socioeconomic status family, problems in emotional control, early difficulties in making friends and parental practices characterized by lower norms. These variables explained almost 50% of the variance of parents and teachers reporting victimization with a near to excellent ability to discriminate between the groups with and without warning signs of potential victimization.

Low SES at age 3 was more frequent in the at-risk group. Several studies showed that being involved in peer victimization either as victim or perpetrator is more frequent in children and adolescents with a lower SES [10,51]. Families with a low SES might have influenced children’s involvement in victimization in several ways. For instance, they tend to have a lower participation in social activities [81]. In low SES families, parents tend to have less involvement in their children’s education, practicing low levels of academic socialization with them [82]. The educational level of parents was included in the family SES calculation because education not only influences economic status, but also reflects non-economic social characteristics, such as intelligence, knowledge, social norms and values, literacy and competence in problem solving [10], all of which are aspects that could be related to child raising behavior and, consequently, to children’s limitations in the development of social skills and coping strategies [10,51]. Families living with this adversity would need victimization surveillance.

Difficulties in emotional control reported by teachers was found to increase the risk of parents and teachers reporting that the child was being picked on or bullied. In other words, children who overreact to small problems, are explosive, have outbursts for little reason, are angry or tearful or react very strongly to situations, are targets for victimization. This result is consistent with previous studies, among them a methodologically similar study with parents and teachers as informant and children from 3 to 5 years showing a high association between emotion dysregulation and higher rates of victimization [83]. This finding agrees with predictions derived from the social process model [84] because emotional dysregulation may cause peers isolation, negative judgments and consequently increase the risk for victimization. This path from emotional control to victimization through social processes was also evidenced in clinical samples of children with a diagnose of attention-deficit/hyperactivity disorder [85] and children with early manifestations of oppositional defiant disorder [46]. Both disorders are characterized by high emotional reactivity, demonstrating that these disorders at preschool age predicted children’s risk of bullying involvement in the first years of elementary school. Children who show high emotional reactivity at preschool entry may therefore require special vigilance at school.

Difficulty in making friends was another strong predictor of parents and teachers reporting that the child was victimized. From early childhood, social competence is an important protective factor for children’s adjustment, helping them to engage in shared activities, adapt to different situations, develop friendships, respect turns, regulate emotionality, collaborate, show prosocial behavior, acquire a good reputation among peers and be accepted by them [86]. Social competence is thus a solid base for building good relationships and when this is not well developed the child is at risk of being isolated and isolated children are also a target for victimization [87].

Regarding parenting practices, our results are in line with previous studies supporting that poor parental supervision, harsh parental discipline, low parental involvement with the child and marital discord are associated with childhood aggression and bullying [88]. Relatively little is known, however, about how this influence operates [89]. A low parental educational level in social norms may interfere with proper socialization as the child has a deficit in references about how to behave with others and this has also been related to victimization in general population [90].

### Limitations

This study has some limitations that must be highlighted. We did not include information reported from the child because the young age of the participants and the methods used hampered their participation. In older children, parents are usually unaware of peer victimization involvement, in part because young people do not always divulge their experiences with victimization to their parents [91], whereas parental follow-up is tighter with preschoolers. Having no information directly from the child, however, could mean that some cases of victimization did not come to light.

A second limitation is that a high percentage of the participant attrition in the follow-up at age 5 was among low socioeconomic status families. This could have resulted in a slight under-estimation of the prevalence of victimization.

Another limitation is in reference to the social-ecological model, because we have not taken into account the community contexts in which social relationships are embedded. The classroom environment had a great influence on the amount of victimization reported by peers, indicating that there is something in the class context that potentiates or inhibits bullying [92].

On the other hand, this study also has several strengths. Peer victimization was assessed using multiple informants (parents and teachers) and considers risk factors from multiple domains (individual and family). Last, much of the previous work on victims has been conducted with older children and there are only a limited number of studies that focus on the preschool period.

### Conclusions

Peer-victimization starts at preschool ages, so early intervention in the preschool context is crucial if we are to break the cycle of victimization in later childhood [34]. Since both individual and family risk factors of different nature are present in the victims, to stop the victimization from escalating requires a broad and multidisciplinary approach.

### Implications for school health

School is a context where peer victimization may occur, and this markedly alters community mental health. The development of programs designed to prevent its escalation, reduce its negative impact on children’s development and promote adjustment are needed. Early identification of both the problem itself, using screening questionnaires for detecting warning signs, and of the problems associated with victimization dynamics may enable closer monitoring of the risk groups as a preventative measure. The prevention of victimization and its consequences can be improved by focusing on risk groups at school in early life. Our results suggest that victimization intervention programs should consider the role of the victims’ personal characteristics in increasing vulnerability to victimization and the importance of including families in school-based intervention programs aimed at reducing the difficulties experienced by victimized children. In summary, research identifying the risk factors for victimization helps to target interventions to deal with the key factors contributing to the problem, thus maximizing their effectiveness [92].
